# Dolphin Morbillivirus Associated with a Mass Stranding of Sperm Whales, Italy

**DOI:** 10.3201/eid2301.160239

**Published:** 2017-01

**Authors:** Sandro Mazzariol, Cinzia Centelleghe, Andrea Di Provvido, Ludovica Di Renzo, Giusy Cardeti, Antonella Cersini, Gianluca Fichi, Antonio Petrella, Cristina Esmeralda Di Francesco, Walter Mignone, Cristina Casalone, Giovanni Di Guardo

**Affiliations:** University of Padova Department of Comparative Biomedicine and Food Science, Padua, Italy (S. Mazzariol, C. Centelleghe);; Istituto Zooprofilattico Sperimentale dell’Abruzzo e Molise, Teramo, Italy (A. Di Provvido, L. Di Renzo);; Istituto Zooprofilattico Sperimentale del Lazio e della Toscana, Rome, Italy (G. Cardeti, A. Cersini);; Istituto Zooprofilattico Sperimentale del Lazio e della Toscana, Pisa, Italy (G. Fichi);; Istituto Zooprofilattico Sperimentale della Puglia e della Basilicata, Foggia, Italy (A. Petrella);; University of Teramo Faculty of Veterinary Medicine, Teramo (G. Di Guardo G., C.E. Di Francesco);; Istituto Zooprofilattico Sperimentale del Piemonte, Liguria e Valle d’Aosta, Imperia, Italy (W. Mignone);; Istituto Zooprofilattico Sperimentale del Piemonte, Liguria e Valle d’Aosta, Turin, Italy (C. Casalone)

**Keywords:** morbillivirus, *Physeter macrocephalus*, mass stranding, dolphins, whales, Italy, H gene, viruses, zoonoses

## Abstract

In September 2014, seven sperm whales were stranded along Italy’s Adriatic coastline. Postmortem investigations on 3 female adult whales and 1 male fetus carried by the largest female revealed molecular and immunohistochemical evidence of dolphin morbillivirus infection. A possible role of the virus in the stranding event was considered.

The mass strandings of sperm whales (*Physeter macrocephalus*) are still largely unexplained events. Solar cycles, weather conditions, coastal geographic features, and human activities have been proposed as possible causes (*1*). Although a multifactorial etiology was hypothesized to be responsible for a mass stranding that occurred in December 2009 involving 7 male sperm whales along the southern Adriatic coast of Italy (*1*), well-defined causes of similar events are rarely identified.

In September 2014, seven sperm whales were found stranded along the central Adriatic Italian coastline; 4 of these whales were refloated. Three females (sperm whale 1 [SW1], pregnant, total length 8.95 m; SW2, total length 8.38 m; and SW3, total length 7.33 m) died on the beach, and postmortem analyses revealed that the largest animal, a pregnant female (SW1), whose male fetus (SW1b) also died, was affected by a prominent hydronephrosis caused by a large stone occupying >50% of the right kidney pelvis. This condition might have resulted in renal function impairment. We observed microscopically evidence of lymphoid cell depletion in several secondary lymphoid tissues from all 3 adult whales, and we found definitive biomolecular evidence of dolphin morbillivirus (DMV) infection in the 3 adult whales and in the fetus.

Our use of 1-step PCR protocols (*2*) failed to sequence any viral fragment, probably because of postmortem changes and subsequent viral RNA degradation by ribonucleases, which progress rapidly in large whales because of their body features and size and thus affect the integrity of the DMV genome. Therefore, we used a more sensitive, nested reverse transcription PCR technique that targeted a highly preserved fragment of the viral hemagglutinin (H) gene (*3*) to investigate the lung (SW1), brain (SW3), and lymphoid tissues (SW2 and SW3) of the 3 adult whales and the lung, kidney, and liver of the fetus (SW1b). We found all of these tissues to be positive for DMV, and we analyzed the gene sequences obtained from the 4 whales’ tissues by using an ad hoc computer program (Lasergene package version SeqMan Pro; DNASTAR Inc., Madison, WI, USA). The hemagglutinin consensus fragment obtained from all the positive samples showed 100% sequence homology with the corresponding DMV genome sequence (GenBank accession no. AJ608288). In addition, we observed simultaneous immunohistochemical (IHC) evidence of DMV nucleoprotein antigen in macrophages and follicular dendritic-like cells from the white pulp of the spleen from the youngest female whale (SW3) and in monocytes circulating within splenic blood vessels from the same animal (Figure). Other target tissues from this whale and the 3 others were either negative or unsuitable for IHC and biomolecular analyses.

**Figure:**
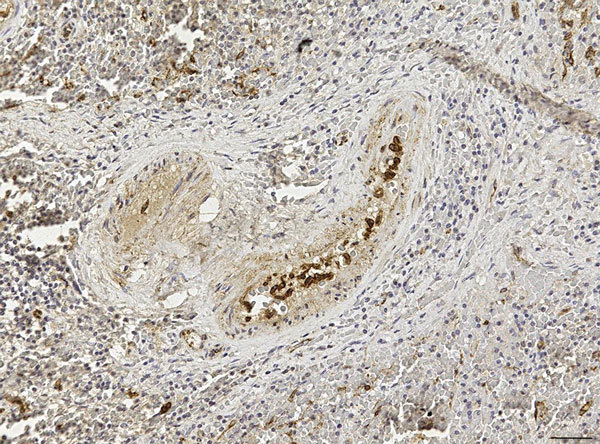
Spleen of the youngest female sperm whale (SW3) in study of 7 sperm whales stranded along Italy’s Adriatic coastline in September 2014. Positive immunostaining (Mayer’s hematoxylin counterstain) for morbilliviral antigen is shown in monocytes within vascular lumina and in follicular dendritic-like cells in the splenic white pulp. Morbillivirus immunohistochemistry was conducted with a murine monoclonal antibody against canine distemper virus nucleoprotein (VMRD Inc., Pullman, WA, USA). Original magnification ×20; scale bar indicates 50 μm.

Although we did not observe any classic DMV-related pathologic changes during postmortem examinations (*2*,*4*), we strongly suspected a viremic condition in all 4 whales on the basis of IHC (SW3) and biomolecular (SW1, SW1b, SW2, and SW3) findings. In this respect, the consistent immunolabeling of morbillivirus antigens in circulating monocytes and in splenic follicular-like dendritic cells support the hypothesis that DMV infection was in an early developmental stage (*5*). Experimental studies conducted on similar morbillivirus infection models show that during this period, even if no severe clinical signs are observed, a general discomfort condition, secondary to viremic circulation, could be reasonably expected (*6*,*7*).

DMV infection has been frequently associated with mass deaths during epidemic outbreaks (*2*); however, it has been seldom reported in single mass stranding events among cetaceans in general, much less among sperm whales. Furthermore, given the documented susceptibility of sperm whales to cetacean morbillivirus and the likelihood of maternal–fetal transfer of the virus (*8*), the biomolecular evidence of DMV infection obtained in the fetal sperm whale in our investigation strongly supports the assumption of a transplacentally acquired infection in this animal.

Although no clear-cut evidence exists that DMV was the primary cause of the mass stranding of sperm whales we report, a vast body of scientific literature is available to support the primary pathogenicity of morbillivirus genus members for their mammalian hosts, including aquatic mammals (*3*). In this respect, previous studies would suggest that chemical pollutants, especially methylmercury, were a relevant causative factor in the stranding and subsequent deaths of the 3 sperm whales in our study (*9*). However, our findings suggest the possible additional role of the DMV infection in this event because it is one of the factors that could explain the whales’ northward trajectory into the Adriatic Sea, which is known to be a cul-de-sac for this species (*1*).

In conclusion, although a vast body of scientific data supports the direct involvement of cetacean morbillivirus clade members such as DMV in the numerous epidemics that have occurred in the last 25–30 years among free-ranging cetaceans worldwide (*2*), their causative role in single mass stranding events cannot be precisely defined. Several additional biologic, ecologic, and environmental factors should be investigated by using a multidisciplinary approach (*10*).
